# Surgical treatment of chondrosarcoma of the sacrum with cement augmentation: A case report

**DOI:** 10.1097/MD.0000000000018413

**Published:** 2019-12-16

**Authors:** Shuzhong Liu, Xi Zhou, An Song, Zhen Huo, Yipeng Wang, Yong Liu

**Affiliations:** a Department of Orthopedic Surgery, Peking Union Medical College Hospital, Peking Union Medical College and Chinese Academy of Medical Sciences; b Department of Endocrinology, Key Laboratory of Endocrinology, National Health and Family Planning Commission, Peking Union Medical College Hospital, Chinese Academy of Medical Science and Peking Union Medical College; c Department of Pathology, Peking Union Medical College Hospital, Chinese Academy of Medical Science and Peking Union Medical College, Beijing, China.

**Keywords:** cement augmentation, diagnosis, sacrum, spinal chondrosarcoma, surgical treatment

## Abstract

**Rationale:**

Chondrosarcoma of the sacrum is a highly unusual disease without standard curative managements yet. The objective of this study is to report a very rare case of chondrosarcoma of the sacrum successfully operated by percutaneous vertebroplasty. The management of these unique cases has yet to be well-documented.

**Patient concerns:**

A 45-year-old woman presented with a five-month history of continuous and progressive pain and numbness of left extremity. A lytic, expanding lesion of the sacrum and paraspinal region with severe epidural spinal cord compression was identified.

**Diagnosis:**

MRI of spine showed spinal cord compression secondary to the epidural componant of the giant mass, with increased marrow infiltration of the left S2 vertebral and paravertebral region, which presented as a solid tumor. Post-operative pathology confirmed the diagnosis of sacral well-differentiated chondrosarcoma (stage I B).

**Interventions:**

The patient underwent percutaneous vertebroplasty and cement augmentation of sacrum via a posterior approach.

**Outcomes:**

The patient's neurological deficits improved significantly after the surgery, but the patient died of multiple systemic metastases at the 2-year follow-up visit. There were no complications associated with the operation during the follow-up period.

**Lessons:**

Taken together, the lesion's clinical features, imaging results, and pathological characteristics are unique. Combined efforts of specialists from orthopedics, radiology, neurosurgery, pathology, and medical oncology led to the successful diagnosis and management of this patient. Giant sacral chondrosarcoma, although rare, should be part of the differential diagnosis when the patient presents with back pain and radiculopathy. We recommend the posterior approach for spinal decompression of the sacral chondrosarcoma when the tumor has caused neurological deficits or other severe symptoms. Osteoplasty by cement augmentation is also a good choice for surgical treatment.

## 1 Introduction

Chondrosarcoma is a malignant tumor comprised of transformed cells producing the cartilaginous matrix without tumor osteoid tissue.^[1]^ Its estimated annual incidence is 1 in 200,000 to 500,000 with 6.5% to 10% of cases arising within the mobile spine and only 5% located within the sacrum.^[1–3]^ Chondrosarcoma may arise in normal bone or may undergo malignant transformation from a previously benign cartilaginous tumor (e.g., enchondroma or osteochondroma).^[1–3]^ Within the whole spine, chondrosarcoma has a predilection for the thoracic spine, but can arise from anywhere along the length from cervical spine to sacrum.^[4,5]^ It typically develops in the posterior elements with extension into the vertebral body (45%) or confined to the posterior elements (40%), with the minority of all cases (15%) confined to the vertebral body.^[1–3]^

To the best of our knowledge, this is a rare case of sacral chondrosarcoma in a woman presenting with radiculopathy, who underwent percutaneous vertebroplasty and cement augmentation. In the follow-up period, the patient's conditions improved significantly postoperatively. After reviewing pertinent literatures, we discussed common perioperative considerations in patients with giant chondrosarcoma of the sacrum and management considerations for these unique cases.

## 2 Case report

In December of 2016, a 45-year-old woman presented to our hospital, with progressive radiating pain and numbness of her left lower limb. In the medical journal of her current illness, the patient stated she had been experiencing a worsening numbness and radiating pain of her left lower limb for approximately five months, and she had also experienced paroxysmal lumbosacral pain for approximately two months. The pain in her left limb can reach 6–7 points using visual analogue scale (VAS) and cannot be alleviated with rest and hot compresses. The patient denied experiencing any other constitutional symptoms. Upon further questioning, she recalled a history of difficulty to control defecation during the past days. No pertinent family history was identified, including hypertension and cancer.

**Figure 1:**
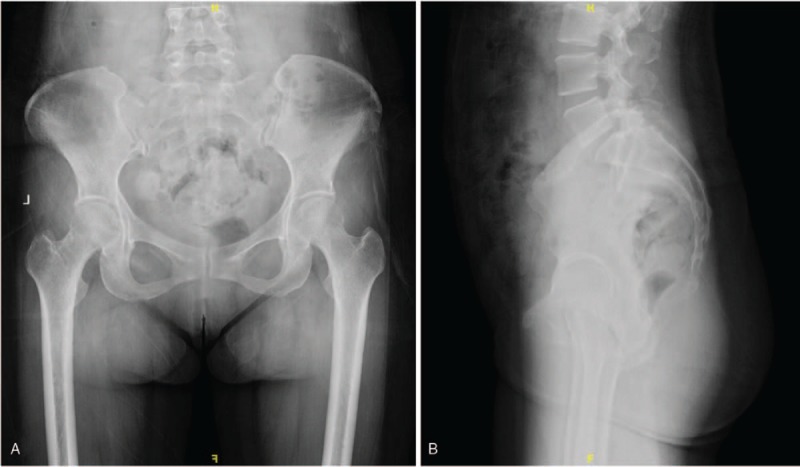
(A, B) Preoperative X-rays revealing sacral lesions with high suspicion of soft tissue tumors.

**Figure 2:**
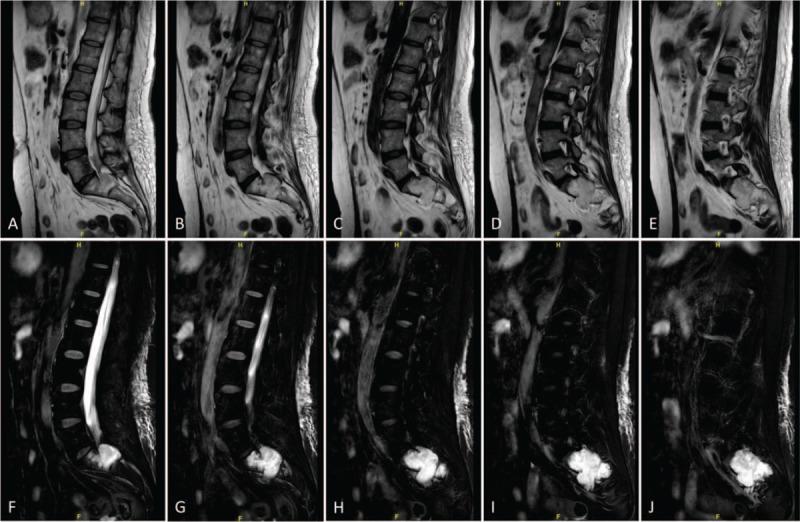
(A–J) Preoperative sagittal MRI scan revealing the density of soft tissue with obvious bony destruction in the sacrum, and significant spinal cord compression secondary to the mass, with increased marrow infiltration of the sacrum.

**Figure 3:**
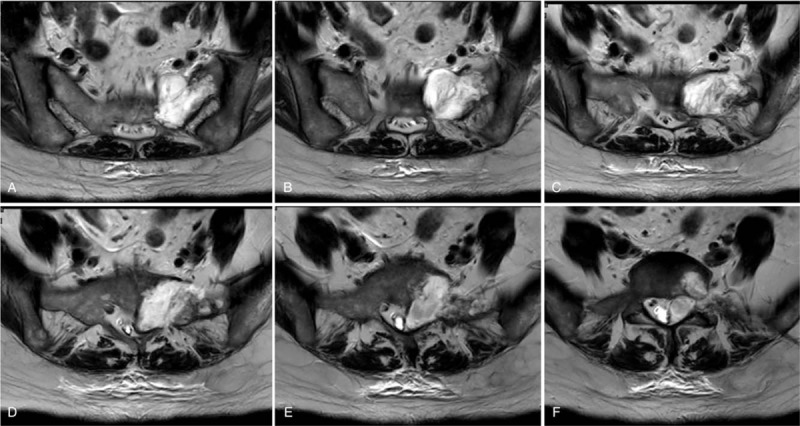
(A–F) Preoperative transverse MRI images showing sacral involvement of chondrosarcoma. Tumor infiltrated through the sacrum body into the posterior elements, resulting in severe spinal cord compression.

On physical exam, the patient showed pressure pain and percussion pain in the sacral region, decreased sensation to pin-prick and fine-touch of her left lower limb and exhibited a 5-/5 strength in her left lower limb. Deep tendon reflexes revealed normal for knee jerk and Achilles tendon reflexes bilaterally. Ataxia, cranial nerves, mini mental, and the rest of the neurological examination showed no abnormalities. Preoperative assessments included electrocardiogram, echocardiogram, and chest radiography. Preoperative laboratory assessment was conducted, including routine laboratory tests (electrolytes, liver and kidney function tests, complete blood count), and tumor markers. The results of the laboratory studies were almost within normal range, except the Carbohydrate antigen 242 levels was mildly elevated to 26.8 U/ml (Normal: 0–20 U/ml). X-rays revealed the shadow of sacral mass, with high suspicion of malignancy (Fig. 1A and B). Spinal MRI was ordered to visualize the sacral lesions, assess the stability of the vertebral column, and to aid in the formulation of a surgical approach. MRI of the spine showed the density of soft tissue, obvious bony destruction in the sacrum, and significant spinal cord compression secondary to the mass, with increased marrow infiltration of the sacrum (Fig. 2A–J). Tumor infiltrated through the sacrum body into the posterior elements, thus extraosseously spread into the left lateral aspect of the epidural space extending posteriorly, resulting in severe spinal cord compression (Fig. 3A–F).

**Figure 4:**
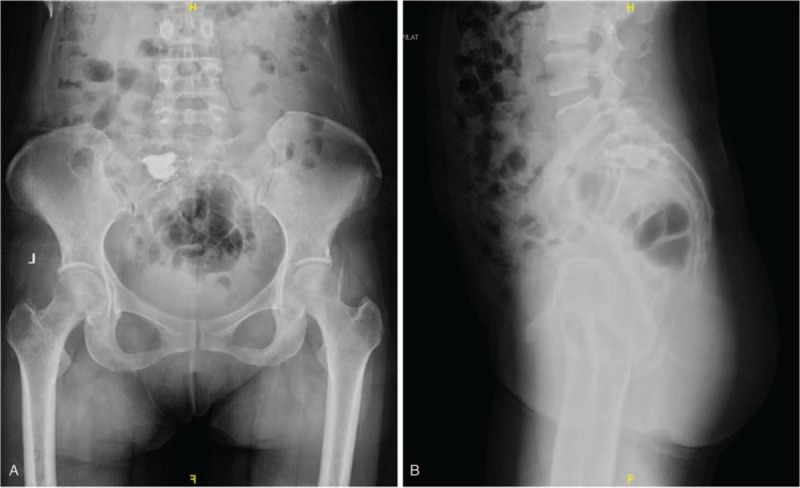
(A) Posteroanterior (PA) x-ray image of the sacrum obtained postoperatively. (B) Lateral x-ray image of the sacral spine obtained postoperatively.

**Figure 5:**
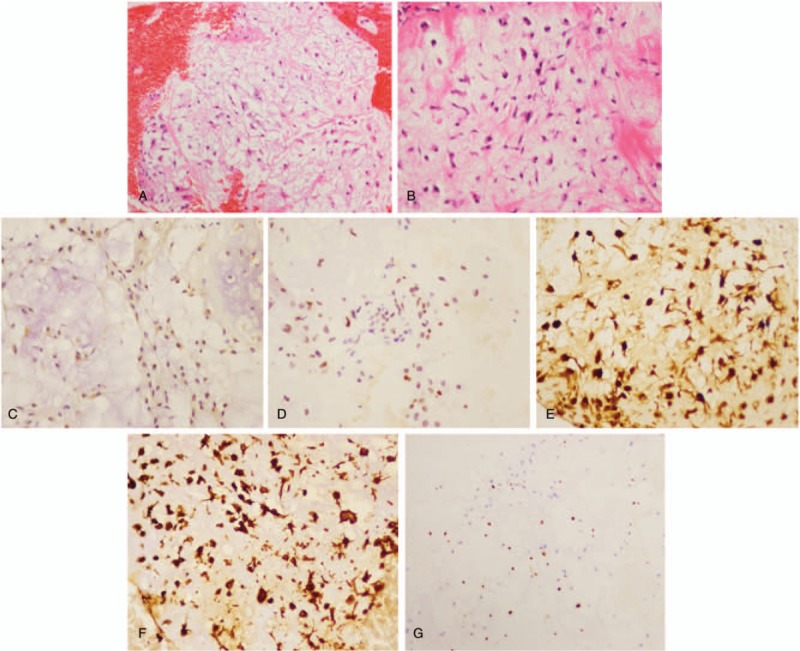
Pathologic histology of sacral chondrosarcoma. (A, B) Microphotography showing characteristic nests of tumor cells separated by vascular septa (Zellballen) with cells showing significant nuclear pleomorphism with prominent nucleoli (H&E, original magnification 100× and 200×). (C) EMA immunostaining is sporadically positive in the cells. (D) P53 immunostaining shows sporadically cytoplasmic staining in the tumor cells. (E) The sustentacular cells of the spinal chondrosarcoma showing characteristic staining of S100. (F) Vimentin immunostaining is positive. (G) Ki-67 immunostaining shows 10% Ki-67 positive cells. Ki-67 staining is localized in the tumor nuclei.

Subsequently, percutaneous vertebroplasty and cement augmentation were performed to destroy the functional tumor and stabilize the spine. In brief, percutaneous osteoplasty in the left sacrum was performed according to the original surgical plan. For the anterior approach, we used C-arm for perspective positioning, the lesion in left sacrum was identified as surgical target, and the puncture point was located. Then 2% lidocaine was used for local infiltration anesthesia, and the puncture needle was inserted through the cannula. Under the C-arm fluoroscopy, the lesion was penetrated through the soft tissues. Then we send out the specimens of the tumor for pathological examination. Bone cement for osteoplasty was introduced. Under the perspective, the 3.2 ml bone cement was slowly pushed through the putter, and the biopsy passage was closed. Fluoroscopy confirmed the good dispersion of bone cement. The operation was successful and intraoperative bleeding was about 30 ml. Postoperative posteroanterior and lateral radiographs of the spine showed cement augmentation was satisfactory (Fig. 4A and B). Histopathologic examination including immunohistochemical staining was performed, and the diagnosis of sacral well-differentiated chondrosarcoma (stage I B) was made according to the criteria (Fig. 5A–G). Pathological analysis was positive for Vimentin, P53, S-100, CD68 and EMA indicating the diagnosis of chondrosarcoma, and biopsy samples were negative for AE1/AE3, CD34, P63, SMA, CgA, Desmin, Myo-D1, with 10% Ki-67 positive nuclei (Fig. 5A–G). Thus, the patient experienced pain relief and improvement of leg numbness. The patient was unwilling to undertake any further treatments and was discharged and monitored on an outpatient basis.

One week after the operation, the patient's muscle strength of left lower extremity improved to grade V compared to the preoperative status, grade V-, and the sensation to pin-prick and fine-touch of left lower extremity returned to normal. Moreover, VAS score of her radiating pain of left lower limb improved to 0–1 points compared to the preoperative status, 6–7 points. Postoperatively, the patient underwent rehabilitation therapy and was discharged and monitored as an outpatient. There were no complications associated with the operation during the follow-up period. However, the patient died of multiple systemic metastases at the 2-year follow-up visit.

## 3 Discussion

Chondrosarcoma (CS) is a cartilage-forming, low-grade malignant neoplasm that accounts for approximately 10% of all bone tumors, with less than 10% of CS involving the spine.^[1–3]^ Few reports of CS involving the spinal region causing clinical symptoms have been documented so far, thus there is yet a consensus on the treatment for CS in the spine.^[4,5]^ Spinal CS can occur primarily or secondarily from an existing cartilage lesion such as an enchondroma or osteochondroma. It more commonly arises in men than in women, with a 3:2 ratio predominantly.^[6,7]^ Typical manifestations include back pain, radiating pain of lower limb, paresthesia, numbness, dysfunction, paraplegia or incomplete paralysis.^[1–3,8,9]^ Among the above symptoms, paroxysmal back pain can often mimic the most common result of other disorders, making timely diagnosis of spinal CS difficult without a high level of suspicion.^[8,9]^ Spinal CS can occur at any level along the spinal axis, although they most commonly present in the thoracic and lumbar regions, often presenting with symptoms of back pain, and spinal cord or nerve root compression.^[8–10]^ The location of the spinal lesion determines the neurological deficits, and there is a great deal of variability.

Radiographically, the tumors are often lytic lesions, presenting with cortical bone destruction. Clinical studies looking at spinal CS is lacking due to the extremely low incidence rate. Imaging studies including CT, MRI, bone scan, and PET/CT are non-specific, making it difficult to differentiate spinal CS from other common spinal disorders.^[11–13]^ However, imaging studies may play a crucial role in the decision making of surgical intervention. The “gold-standard” diagnosis of spinal chondrosarcoma relies on pathological findings.^[14,15]^

Surgery is the best treatment for spinal CS causing back pain, radiculopathy, and paralysis.^[16–18]^ This protocol enables accomplishment of 2 objectives: it alleviates the neurological deficits by decompressing the stenosis while provides histopathological specimens for diagnosis at the same time.^[19]^ Nevertheless, there are several considerations to be kept in mind when deliberating on surgical intervention to CS with spinal involvement, including preoperative spinal instability and selection of operative procedures, possible incomplete tumor resection, intraoperative blood loss and protecting the nerves and vessels, as well as postoperative adjuvant therapy.^[19–22]^

To date, surgical management of CS of spine has remained under evaluation, with no standard criteria. No systematic review comparing patient outcomes and surgery types in primary spinal CS has been conducted based on our literature review. Extent of surgical resection is reported to be correlated with overall survival benefit, and en bloc tumor resection with spinal stabilization is the gold standard of surgical treatment. Osteoplasty by cement augmentation may be a treatment option for patients with CS in the spine, who cannot undergo appropriate surgery or decline open surgery.^[20,21]^ However, we need to fully recognize the potential risk of complications in bone cement applications. The safety of this approach still needs to be confirmed in further studies with larger sample sizes and longer follow-up periods. One postoperative complication was cement leakage into the canal and subsequent spinal cord compression.^[20–22]^ Under this circumstance, surgical extent, cement volume, and postoperative complications are critical factors that need further investigation.^[20–22]^

The survival benefit of resection of primary spinal CS is still unproven. However, such a procedure does have the benefit aiming at controlling residual tumor.^[1–3,23,24]^ The improved survival benefited from reducing the tumor burden, decompressing the spinal stenosis to alleviate radiculopathy, and facilitating subsequent chemotherapy and radiation therapy.^[25–27]^ Due to its rarity, the chemotherapy and radiotherapy regimes have not reached a concensus. A main feature of spinal CS is not sensitive to both chemotherapy and radiotherapy, thus patients suffering from spinal CS with complex conditions and advanced stages may receive chemotherapy and radiotherapy.^[28–31]^ Moreover, recurrence and metastasis are common postoperative complications due to its invasive nature, and metastases occured postoperatively in our patient which lead to her death. They account for a significant percentage of morbidity following resection of CS in the spine.^[30,31]^

## 4 Conclusion

In conclusion, we expect that this report can draw the attention from the research community to how we diagnosed and managed a patient with sacral CS. Although uncommon, sacral CS should be part of the differential diagnosis when the patient presents with back pain and neurological deficits, and pathological examination remain the “gold standard” for diagnosing sacral CS. Moreover, osteoplasty by cement augmentation is also a good choice for surgical treatment. However, we need to take the potential risk of complications in bone cement applications into full consideration. With a multidisciplinary team approach, proper planning, and adequate perioperative medical management, chondrosarcoma in the spine can be managed much more effectively.

## Acknowledgments

We would like to thank our colleagues at the Department of Orthopedic Surgery, Peking Union Medical College Hospital, Chinese Academy of Medical Sciences and Peking Union Medical College.

## Author contributions

**Conceptualization:** Shuzhong Liu, Xi Zhou, An Song, Yipeng Wang, Yong Liu.

**Funding acquisition:** Shuzhong Liu, Yipeng Wang, Yong Liu.

**Investigation:** Shuzhong Liu, Xi Zhou, Yong Liu.

**Resources:** Shuzhong Liu, Xi Zhou, Zhen Huo, Yong Liu.

**Supervision:** Yipeng Wang, Yong Liu.

**Writing – original draft:** Shuzhong Liu, Xi Zhou, An Song.

**Writing – review & editing:** Shuzhong Liu, An Song, Yipeng Wang, Yong Liu.
